# A Metabolic Profiling Analysis Revealed a Primary Metabolism Reprogramming in Arabidopsis *glyI4* Loss-of-Function Mutant

**DOI:** 10.3390/plants10112464

**Published:** 2021-11-15

**Authors:** Silvia Proietti, Laura Bertini, Gaia Salvatore Falconieri, Ivan Baccelli, Anna Maria Timperio, Carla Caruso

**Affiliations:** 1 Department of Ecological and Biological Sciences, University of Tuscia, 01100 Viterbo, Italy; s.proietti@unitus.it (S.P.); lbertini@unitus.it (L.B.); gfalconieri@unitus.it (G.S.F.); 2 Institute for Sustainable Plant Protection, National Research Council of Italy, Sesto Fiorentino, 50019 Florence, Italy; ivan.baccelli@ipsp.cnr.it

**Keywords:** methylglyoxal, glyoxalase I, metabolite profiling, oxidative stress, plant growth, plant defense

## Abstract

Methylglyoxal (MG) is a cytotoxic compound often produced as a side product of metabolic processes such as glycolysis, lipid peroxidation, and photosynthesis. MG is mainly scavenged by the glyoxalase system, a two-step pathway, in which the coordinate activity of GLYI and GLYII transforms it into D-lactate, releasing GSH. In *Arabidopsis thaliana*, a member of the GLYI family named GLYI4 has been recently characterized. In *glyI4* mutant plants, a general stress phenotype characterized by compromised MG scavenging, accumulation of reactive oxygen species (ROS), stomatal closure, and reduced fitness was observed. In order to shed some light on the impact of *gly4* loss-of-function on plant metabolism, we applied a high resolution mass spectrometry-based metabolomic approach to Arabidopsis Col-8 wild type and *glyI4* mutant plants. A compound library containing a total of 70 metabolites, differentially synthesized in *glyI4* compared to Col-8, was obtained. Pathway analysis of the identified compounds showed that the upregulated pathways are mainly involved in redox reactions and cellular energy maintenance, and those downregulated in plant defense and growth. These results improved our understanding of the impacts of *glyI4* loss-of-function on the general reprogramming of the plant’s metabolic landscape as a strategy for surviving under adverse physiological conditions.

## 1. Introduction

During evolution, plants have evolved sophisticated mechanisms allowing adaptation and survival under biotic and abiotic stress conditions [1,2]. After sensing the stimulus, plants activate general reprogramming of the genetic machinery and of the metabolic profile, leading to efficient defense responses and ultimately to plant tolerance [2]. The finely tuned defense mechanisms are substantially orchestrated by small molecules, such as phytohormones, plant volatile organic compounds (VOCs), and primary and secondary metabolites [3,4,5]. Besides, plants exposed to stresses often produce toxic compounds, such as methylglyoxal (MG)—one of the most ubiquitous toxins produced [6,7,8,9,10]. MG is a reactive alpha-ketoaldehyde formed as a side product of metabolic processes such as glycolysis, lipid peroxidation, photosynthesis, and protein degradation [11]. In particular, a high concentration of MG affects seed germination, plant growth and development, and photosynthesis [11,12]. It can also regulate stomatal closure by triggering accumulation of reactive oxygen species (ROS) in the guard cells [13]. Noteworthy, MG spontaneously glycates nucleic acids, proteins, and lipids, forming advanced glycation end products (AGEs) that are involved in oxidative stress onset and can alter the expression of signal transduction-related genes involved in cellular metabolism and transport, protein degradation, and stress/defense responses [11]. To limit the damage caused by MG, an important detoxification mechanism ensured by the glyoxalase (GLY) system has evolved. In particular, the MG detoxification process is mostly mediated by two enzymes: glyoxalase I (GLYI) and glyoxalase II (GLYII). GLYI leads to the formation of S-lactoylglutathione, which is subsequently transformed into D-lactate by GLYII, regenerating GSH in the process [13]. By facilitating MG detoxification, plant glyoxalases can be conveniently assigned to diverse physiological roles, which are involved in the detox central pathway for sugar metabolism and other primary physiological processes [12]. In plant species such as rice, tobacco, and tomato the overexpression of glyoxalase genes, either individually (*GLYI* or *GLYII*) or as a complete pathway (*GLYI* + *GLYII*), lead to plants with improved tolerance of biotic, abiotic, and oxidative stresses [12,14,15,16]. Several genes belonging to the GLYI and GLYII families have been isolated and characterized in many plants. For instance, 11 *GLYI* genes, members of the vicinal oxygen chelate (VOC) superfamily, were characterized in Arabidopsis [17]. Recently, we thoroughly studied AtGLYI4, a member of the Arabidopsis GLYI family. A genome-wide association (GWA) study suggested glyoxalase AtGLYI4 as a novel player in defense hormone signaling and in defense against biotic stresses in Arabidopsis [18,19]. Moreover, it has been shown that AtGLYI4 has a great impact on MG scavenging and on plant health. In fact, in the T-DNA insertion line *glyI4*, compromised MG scavenging, ROS accumulation, stomatal closure, and reduced plant fitness were observed [19]. Additionally, accumulation of MG led to lower efficiency of the jasmonate signaling pathway and increased the susceptibility of *glyI4* plants to the necrotrophic fungus *Plectospherella cucumerina* [19].

Under adverse conditions, general reprogramming of signaling and defense responses is widely recognized [20]. Moreover, it is reasonable that plants execute metabolic rewiring too, in order to restore chemical and energetic balances essential for acclimation and survival. Therefore, metabolic profiling analysis can be a valid approach through which to accurately understand the different processes that take place in the plant during stressful conditions. Indeed, metabolites are the results of both biological and environmental factors, and provide great potential to bridge our knowledge about genotypes and phenotypes. Many studies revealed that metabolic activities respond to stress faster than transcriptional activities, thereby making metabolic changes an important component of early stress responses [21]. Moreover, metabolomic approaches are useful to discover novel genes or new gene functions and to characterize the metabolic responses to stress conditions [20]. Finally, the idea that untargeted mass spectrometry (MS)-based metabolomic analysis will result in a large list of identified small molecules that can be mapped to networks and pathways is often assumed [22].

In this work, we aimed to investigate the metabolic profiling of a *glyI4* loss-of-function mutant against Col-8 wild type to unravel the blend of metabolites and the pathways they are involved in, which could further explain the impacts of *glyI4* loss-of-function on the altered physiological processes that we previously observed. Results of LC-MS/MS analysis showed differentially expressed metabolites in *glyI4* mutant with respect to Col-8. Pathway enrichment analysis revealed that the upregulated pathways are mainly involved in redox reactions and in cellular energy maintenance, while the downregulated ones in plant growth and defense. Furthermore, downregulation of marker genes involved in plant growth and defense, such as *PAL2*, *CAD4*, and *EPSPS*, was observed in the *glyI4* mutant. Finally, focusing on redox reactions, enzymatic assays confirmed the impairing of ROS scavenging in the *glyI4* mutant. These results corroborated the role of GLYI4 in the trade-off between plant survival and defense.

## 2. Results and Discussion

### 2.1. GLYI4 Knock-Down Affects Plant Metabolism

**Figure 1:**
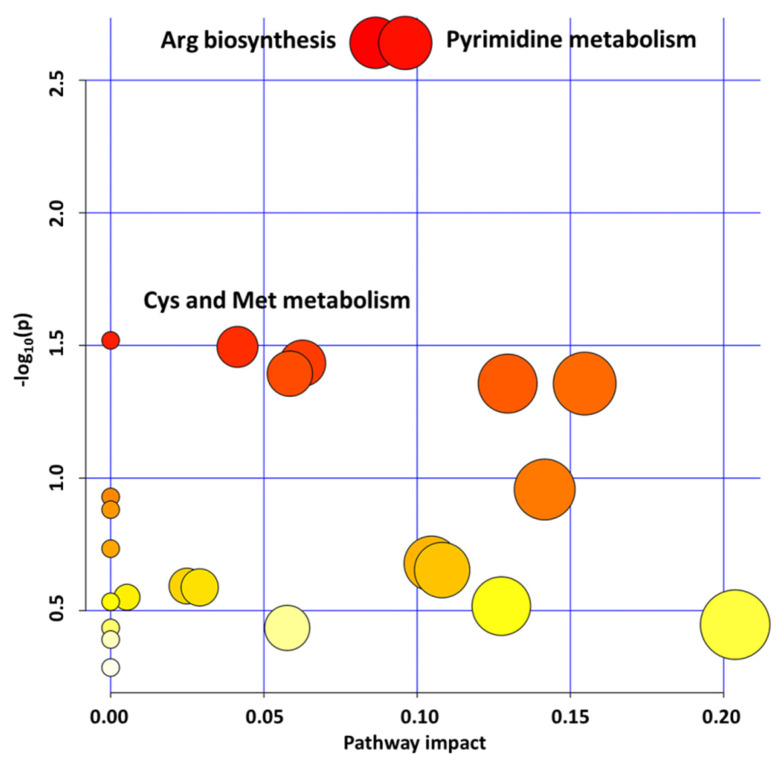
Metabolic pathway analysis plot. Color intensity (white to red) reflects increasing statistical significance, and circle diameter varies with the pathway impact. The graph was made by plotting on the *y*-axis the −log_10_ transforms of *p*-values from the pathway enrichment analysis and on the *x*-axis the pathway impact values derived from the pathway topology analysis. The top three most significant pathways (*p*-value < 0.05; pathway impact (pi) > 0) are depicted: arginine biosynthesis (*p*-value = 0.0022868, pi = 0.08641); pyrimidine metabolism (*p*-value = 0.0022904, pi = 0.09611); cysteine and methionine metabolism (*p*-value = 0.032033, pi = 0.04138). The metabolomic analysis was repeated three times using different biological samples giving the same results.

In the present study, we performed high-throughput metabolic profiling analysis through LC-MS/MS, which allowed us to identify 131 metabolites, among which 70 were differentially synthesized (log_2_-fold change > |1|, *p*-value < 0.05). In detail, 23 of them were over-produced and 47 were under-produced in the glyI4 mutant compared to Col-8 (Supplementary Table S1). The metabolome overview obtained through the Metaboanalyst 5.0 “pathway analysis” tool showed “arginine biosynthesis,” “pyrimidine metabolism,” and “cysteine and methionine metabolism” pathways as the three most upregulated metabolic pathways in glyI4 compared to Col-8, based on *p*-value < 0.05 and pathway impact > 0 (Figure 1).

Moreover, among the first top-ten significantly uniquely enriched pathways were “citrate cycle (TCA cycle),” “carbon fixation in photosynthetic organisms,” “alanine, aspartate and glutamate metabolism,” “pyruvate metabolism,” “glycerophospholipid metabolism,” and “monobactam biosynthesis”.

#### 2.1.1. Pyrimidine Metabolism Upregulation Influences Redox Homeostasis

MG has been found to affect pyrimidine metabolism in human cells [23], whereas in plants its effect on nucleotides biological processes is still unknown.

Nucleobases, such as pyrimidines and purines, are important nitrogen compounds constituting the building blocks necessary for DNA and RNA synthesis; energy sources; coenzymes for redox reactions; and precursors for the synthesis of primary and secondary products, such as isobarbituric acid, glycosides, and β-alanine [24]. Furthermore, pyrimidines play a key role as regulators of amino acid, phospholipid, glycolipid, sugar, and polysaccharide biosynthesis [25]. Pyrimidines are synthetized from amino acids, and small molecules through de novo pathways and from preformed nucleobases and nucleosides through salvage pathways [26]. Since they have important functions in a multitude of biochemical and developmental processes, they are essential during the entire life cycle of the plant. Among all metabolites of pyrimidine metabolism pathway, our attention has been focused on the dihydroorotic acid, which is involved in redox reactions. This metabolite appears to be localized at the mitochondrial level, and this is very interesting, since mitochondria are essential regulators of redox-dependent processes [27]. In fact, in this cellular compartment, redox-sensitive processes occur, providing a rapid response to metabolic changes and reactive oxygen species (ROS) fluxes [28]. The over-production of this metabolite in the *glyI4* mutant could be linked to the previously observed high ROS production in this mutant [19]. Pyrimidine metabolism is also involved in the β-alanine biosynthesis, which was another upregulated pathway in the *glyI4* mutant, although its impact value was below the significant threshold.

#### 2.1.2. Aminoacid Metabolism Upregulation Promotes Cellular Energy Maintenance

It has been proved that MG impacts the metabolism of amino acids in rat cerebral tissues [29]. In our work, we found that among the most significant perturbed pathways are those related to “arginine biosynthesis” and “cysteine and methionine metabolism.” In most terrestrial habitats, nitrogen is a limiting resource for plant growth, since large amounts of nitrogen are needed to synthesize proteins and nucleic acids. Among the 21 proteinogenic amino acids, arginine has the highest nitrogen/carbon ratio, which makes it especially suitable as a form of organic nitrogen storage. Biosynthesis in chloroplasts via ornithine is apparently the only pathway to provide arginine in plants, and the rate of arginine synthesis is tightly regulated by various feedback mechanisms in accordance with the overall nutritional status [30]. Although several steps of arginine biosynthesis still remain poorly characterized in plants, its role in maintaining the energy status of the cell is apparent [31]. Cysteine and methionine metabolism plays a significant role in the biosynthesis of different biomolecules, such as glutathione, ethylene, phytochelatines, polyamines, and biotin [32]. In particular, cysteine is crucial for many biosynthetic pathways, and it is a precursor molecule of diverse sulfur-containing metabolites [33]. In general, cysteine is the direct or indirect donor of all reduced sulfur groups in plant cells. In Arabidopsis, the most important ones are methionine, iron–sulfur cluster, glutathione, molybdenum cofactor, vitamins (coenzyme A, lipoic acid, thiamine, biotin), and secondary compounds such as camalexin and glucosinolates [34]. It has been already reported elsewhere that to cope with a stress, glyoxylases are essential to decrease the methylglyoxal level and increase reduced glutathione and cysteine [35]. In our previous work, we observed deprivation of GSH in *glyI4* as an effect of impaired methylglyoxal detoxification [19]. Interestingly, in our metabolite dataset, we found cystine (Supplementary Table S1). Taken together, the fact that in our model system we observed a GSH decrease and cystine enrichment in *glyI4* could be a sign of the compromised plant health due to MG accumulation. Hence, the results of metabolomic analysis strengthen our previous findings, by using a different approach. Methionine is another important amino acid, belonging to the aspartate family that is involved in plant metabolism, and being part of the protein structure. In Arabidopsis, recent studies have shown that methionine affects the metabolic regulation of other pathways, such as polyamines, ethylene, and glucosinolate pathways [36]. In particular, methionine is synthesized by the aspartate family pathway which leads to the formation of other amino acids, such as threonine and isoleucine. These amino, acids take part in protein synthesis, and additionally, they can be catabolized into the TCA cycle in order to contribute to cellular energy metabolism [37]. Altogether, these results led us to speculate that a general reprogramming towards energy-generating processes could occur in the *glyI4* mutant, allowing the plant to have more energy to adapt, survive, and cope with stressful conditions.

#### 2.1.3. Purine, Aromatic Amino Acid, and Zeatin Biosynthesis Downregulation Affects *glyI4* Mutant Growth and Defense

**Figure 2:**
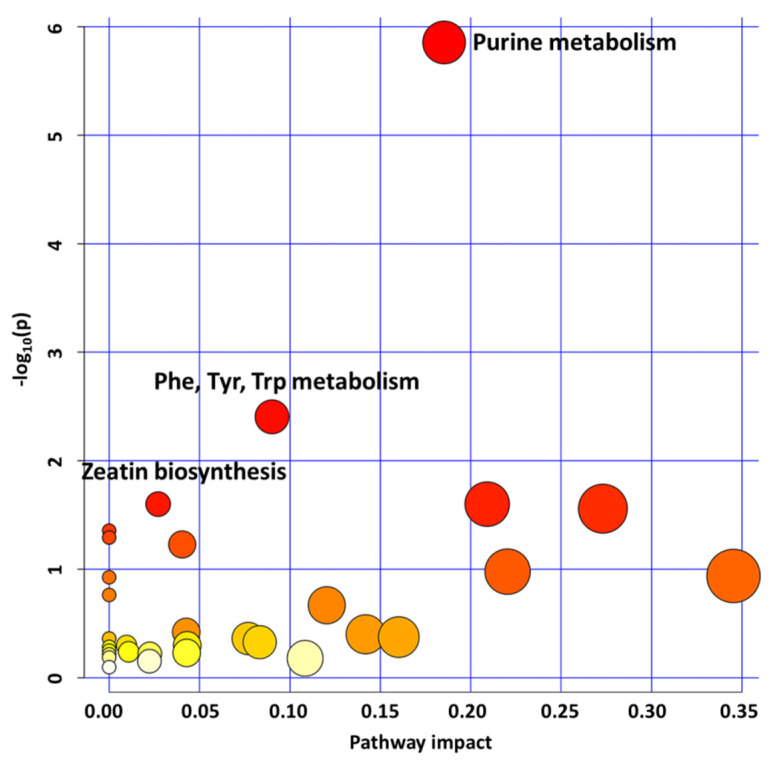
Metabolic pathway analysis plot. Color intensity (white to red) reflects increasing statistical significance, and circle diameter varies with the pathway impact. The graph was obtained by plotting on the *y*-axis the −log_10_ transforms of *p*-values from the pathway enrichment analysis and on the *x*-axis the pathway impact values derived from the pathway topology analysis. The top three most significant pathways (*p*-value < 0.05; pathway impact (pi) > 0) are depicted: purine metabolism (*p*-value = 1.3987 × 10^−6^, pi = 0.18529); phenylalanine, tyrosine, and tryptophan biosynthesis (*p*-value = 0.0039386, pi = 0.09009); zeatin biosynthesis (*p*-value = 0.025118; pi = 0.0271). The metabolomic analysis was repeated three times using different biological samples giving the same results.

We also analyzed the enriched pathways in the under-produced metabolites dataset in the *glyI4* mutant compared to Col-8. As shown in Figure 2, we focused our attention on the top three most significant enriched pathways: “purine metabolism,” “phenylalanine, tyrosine, tryptophan metabolism,” and “zeatin biosynthesis”.

Moreover, among the first top-ten significantly uniquely enriched pathways were “nicotinate and nicotinamide metabolism,” “arginine biosynthesis,” and “pentose phosphate pathway”.

Purines are crucial components of nucleic acids and they play also an important role in metabolic regulation and energy storage. In plants, purines are also involved in nitrogen metabolism and in the formation of metabolic intermediates, among which is allantoin, that could also function in stress protection [38]. Studies in several plant species emphasized the correlation between endogenous allantoin levels and physiological responses to various stress, such as drought [39], nutrient deprivation [40] and high salinity [41]. Moreover, allantoin, and purines in general, enhance stress tolerance and protection by stimulating intermediate metabolites involved in stress responses [42]. Noteworthily, related to the fact that we found “purine metabolism” downregulated while “pyrimidine metabolism” was upregulated, we can mention that there are several endogenous and exogenous factors that affect the synthesis of purine and pyrimidine [26], and the accumulation of methylglyoxal could be one of the causes of the different behavior we found for the two pathways. In addition, we have also to consider that besides being the building blocks for DNA and RNA synthesis, purine and pyrimidine, are also precursors of energy sources, coenzymes, and primary and secondary compounds, whose syntheses can be up and/or downregulated depending on the contingent needs. Moreover, there are examples in the literature where the two pathways do not follow the same trend, supporting the reliability of the result [43]. Purines act also as precursors in the formation of zeatin, which plays a role in cell division, shoot formation, and adaptation to stress, as we will discuss later. Among the downregulated pathways is that of the aromatic amino acids phenylalanine, tyrosine, and tryptophan, which act as precursors of several secondary metabolites important for plant growth. Several lines of evidence suggest that a close relationship exists between cellular redox state and amino acid metabolism—in particular, that related to aromatic amino acids [44]. These amino acids are synthesized via the shikimate pathway and are essential precursors of the phenylpropanoid pathway, which is a great source of metabolites in plants [45]. Phenylpropanoids are required for diverse processes, among which are the lignin biosynthesis and flavonoid and coumarin formation [46]. Recently, flavonoids have been identified as regulators of GLYI and GLYII activity in tomato seedlings under salt stress [47]. In Arabidopsis, the phenylpropanoid pathway has been studied, especially for its correlation in defense and survival mechanisms [45]. These results could be linked to the compromised growth and defense in *glyI4* mutant, as we previously observed [19]. Finally, another downregulated pathway highlighted in this study is that of zeatin biosynthesis. Zeatin is a phytohormone derived from purine metabolism, and it is a component of the family known as cytokinins (CKs). Several studies have shown that cytokinins have roles in plant growth, abiotic stress responses, pathogen and herbivore resistance, and cell division regulation [48]. A great correlation between GLYI and CKs has been documented in several species [47]. A genome-wide transcriptional study of *A. thaliana ipt* mutants (deficient in cytokinin) reported that *GLYI* expression is altered in mutants under stress. In particular, *GLYI4* was fivefold upregulated in mutants versus wild-type under salt stress, probably due to a decreased activity of cytokinin [49]. In addition, it has been reported that a stunted growth occurs in stress conditions, due to the downregulation of the cytokinin pathway [50]. Therefore, this finding could also be linked to the reduced plant development and growth observed in the *glyI4* mutant [19].

Taken together, the metabolite analysis highlighted that when GLYI4 is no longer functional, mechanisms related to plant growth and defense are likely to be affected, and metabolic cellular rewiring involves energy maintenance and redox-related processes.

### 2.2. GLYI4 as a Potential Positive Regulator of Plant Growth and Defense

**Figure 3:**
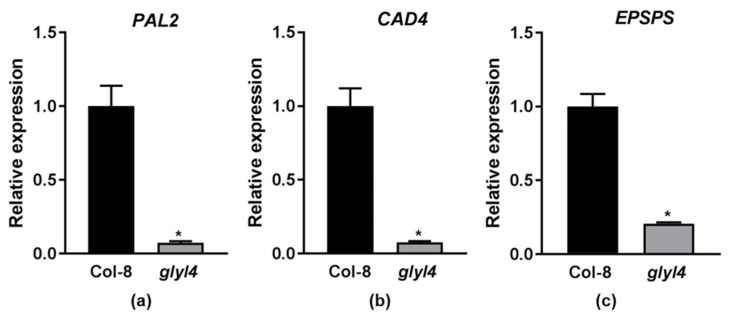
*PAL2* (**a**), *CAD4* (**b**), and *EPSPS* (**c**) transcript levels relative to the reference gene *UBI10* in Col-8 and the *glyI4* mutant. An asterisk indicates a statistically significant difference between genotypes (Mann–Whitney test *p*-value < 0.05). Error bars represent means ± SDs (*n* = 3).

The plant growth–defense trade-off is fundamental for optimizing plant performance and fitness in a changing biotic/abiotic environment. Looking at the results discussed in the previous paragraph, we found very intriguing the link between the downregulation of aromatic amino acids pathway and the shikimate/phenylpropanoid pathway, which is largely implicated in growth and defense. We then decided to check the expression of three valuable marker genes of the shikimate/phenylpropanoid pathway, phenylalanine ammonia lyase 2 (*PAL2*, At3g53260), cinnamyl alcohol dehydrogenase 4 (*CAD4*, At3g19450), and 5-enolpyruvylshikimate-3-phosphate synthase (*EPSPS*, At1g48860), in Col-8 and the *glyI4* mutant. As shown in Figure 3, the three genes were significantly downregulated in the *glyI4* mutant compared to Col-8.

It has frequently been observed that among secondary cell wall components, alterations in lignin biosynthesis result in changes in both growth and defense [51]. Lignin is an aromatic heteropolymer synthesized through an intricate phenylpropanoid metabolism. The phenylpropanoid pathway starts with the deamination of L-phenylalanine into cinnamic acid by PAL enzymes. The cinnamic acid undergoes a series of reduction processes in which the CAD enzymes are involved, to finally produce monolignols whose polymerization yields lignin [52]. Lignin is the second major structural component of the plant cell wall in supporting and conducting tissues, and it is used to reinforce mechanical strength and create a chemical and microbial resistant barrier [53]. Lignin, together with other chemical compounds such as suberin and phenols, is the first barrier in case of pathogen penetration [54,55,56,57]. For instance, host cell-induced lignin biosynthesis in response to fungal colonization has been reported in banana roots [58], wheat cells [59], and *Camelina sativa* [52,60]. In Arabidopsis, *CAD*, and *PAL* genes have been found to act as essential components of defense against virulent and avirulent strains of the bacterial pathogen *Pseudomonas syringae* pv. tomato and as critical component in the resistance against abiotic stresses [61,62,63]. EPSPS catalyzes the transfer of the enolpyruvyl moiety of phosphoenolpyruvate (PEP) to the 5-hydroxyl of shikimate-3-phosphate (S3P) to produce enolpyruvyl shikimate-3-phosphate and inorganic phosphate. EPSPS is a key enzyme in the shikimate pathway, which is extremely important because about 35% or more plant biomass in dry matter form is represented by aromatic molecules derived directly from this pathway [64]. EPSPS is essential for the biosynthesis of aromatic amino acids, lignin, and secondary metabolites, including defensive compounds [65], suggesting that EPSPS is important for the survival, defense, growth, and development of plants. The involvement of this gene in plant growth is further corroborated by the evidence that Arabidopsis plants overexpressing *EPSPS* showed stimulated biosynthesis of auxin, an important plant growth hormone [66]. According to the stunted phenotype of *glyI4* mutant, we can hypothesize that the downregulation of *PAL2*, *CAD4*, and *EPSPS*, compared to Col-8, could be one of the reasons for the impaired plant growth and defense [19].

### 2.3. GLYI4 Affects Plant Antioxidant Responses

**Figure 4:**
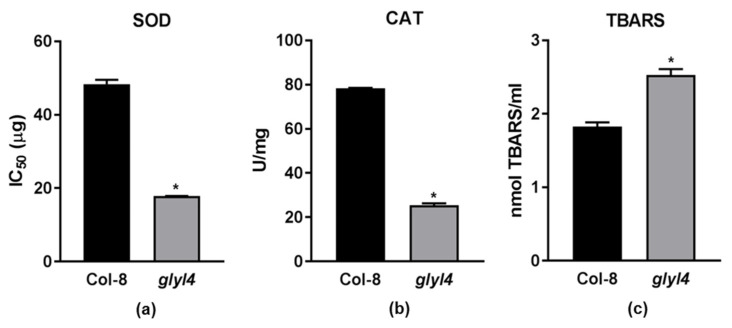
Antioxidant enzyme activities and lipid peroxidation in the *glyI4* mutant. (**a**) superoxide dismutase (SOD); (**b**) catalase (CAT); (**c**) TBARS. Asterisks indicate statistically significant difference between genotypes. (Mann–Whitney test *p*-value < 0.05). Error bars represent means ± SDs (*n* = 3).

ROS are produced in both unstressed and stressed cells. Plants have a well-developed defense systems against ROS, involving both limiting ROS formation and removing them. Under unstressed conditions, the formation and removal of ROS are well balanced. However, the defense system, when dealing with increased ROS formation under stressed conditions, can be overwhelmed. When this occurs, plants respond to high levels of ROS with increased enzymatic or non-enzymatic antioxidant processes [67]. In our previous work, we reported that *glyI4* loss-of-function causes a great increase in ROS production and deprivation of GSH, likely due to MG accumulation [19]. In particular, using a ROS-sensitive dye, a high level of H_2_O_2_ in Arabidopsis *glyI4* leaves was highlighted [19]. To corroborate our previous results and to further validate the impact of *glyI4* loss-of-function on redox processes, we investigated key components of the ROS pathway in the *glyI4* mutant. In particular, we analyzed the activity of ROS scavenging enzymes such as superoxide dismutase (SOD) and catalase (CAT) and lipid peroxidation (by measuring thiobarbituric acid reactive substance assay (TBARS) content) in *glyI4* leaves (Figure 4).

#### 2.3.1. Hydrogen Peroxide Is Not Efficiently Scavenged by Antioxidant Machinery

Within a cell, the SODs constitute the first line of defense against ROS. The superoxide anion (O_2_^−^) is produced at any location where an electron transport chain is active, and hence O_2_ transformation may occur in different compartments of the cell [68], including mitochondria, chloroplasts, microsomes, glyoxysomes, peroxisomes, apoplasts, and the cytosol. Thus, it is not surprising to find that SODs are present in all these subcellular locations [67]. These enzymes belong to the family of metal-enzymes that catalyze the dismutation of superoxide anions (O_2_^−^) into molecular oxygen (O_2_) and hydrogen peroxide (H_2_O_2_). In our work, the IC50 of the *glyI4* mutant was 2.5 times less than that of Col-8, suggesting that less protein extract is required to achieve 50% inhibition of formazan production. This means that SOD activity in the *glyI4* mutant is greater than that in Col-8. High level of SOD activity was found in *Citrus sinensis* leaves where MG accumulation was reported [69]. Catalases (CATs) are heme-containing enzymes present in all organisms that play a central role in maintaining a balance of cellular hydrogen peroxide. The typical catalase reaction is the dismutation of two molecules of H_2_O_2_ to water and O_2_. As H_2_O_2_ is relatively stable and present in several subcellular compartments, its impact is strongly dependent by the removal ability of the antioxidant system [70]. In our work, CAT activity decreased in the *glyI4* mutant as compared to Col-8. Similarly, lower CAT protein levels were found in *Citrus sinensis* leaves that accumulated MG, compared to leaves where MG accumulation was lower. This might be related to the high sensitivity of CAT to oxidative stress [71,72] and to a reduced enzyme production under stress which keeps CAT levels low [69]. According to our results, we can speculate that H_2_O_2_ production driven by SOD is not scavenged by CAT, whose activity is compromised by MG accumulation.

#### 2.3.2. *glyI4* Mutant Suffers of Lipid Peroxidation

Lipid peroxidation is a deleterious process in plants, which affects membrane properties, causes protein degradation, and limits ion transport capacity, ultimately triggering the cell death process [73]. One of the most commonly used methods to detect lipid peroxidation is the TBARS assay. The assay involves the reaction of lipid peroxidation products, primarily malondialdehyde (MDA), with thiobarbituric acid (TBA), which leads to the formation of MDA-TBA2 adducts called TBARS. TBARS produce a red-pink color that can be measured spectrophotometrically at 532 nm. The highest TBARS content was found in the *glyI4* mutant, suggesting a higher level of oxidative stress in these plants, probably due to an impaired detoxification system. In addition, it has been reported that over-accumulation of MG can lead to lipid peroxidation in plant cells [35,74]. In particular, Yadav et al. [35] reported that the salt-induced increases of MG and MDA concentrations were much lower in transgenic tobacco plants overexpressing *GLYI* and/or *GLYII* than in wild-type plants. Taken together, our results corroborate the great impact of MG accumulation on ROS accumulation and scavenging.

## 3. Materials and Methods

### 3.1. Plant Material and Growth Conditions

The *A. thaliana* T-DNA line in Col-8 background *glyI4* (AGI: At1g15380) was purchased from NASC (http://arabidopsis.info/, 20 August 2014), available with the ID: SALK_067593C. The same mutant was already tested and used for experiments [18,19]. Arabidopsis *glyI4* and Col-8 seeds were sown in cultivation containers filled with autoclaved river sand. Sand was supplied with half-strength Hoagland solution (Sigma, Steinheim, Germany). To achieve a high relative humidity for germination, cultivation containers were enclosed in a tray with water and covered with a transparent lid. Seeds were stratified for 2 days at 4 °C in the dark to ensure a homogeneous germination after which the tray was moved to a growth chamber with an 8-h day/16-h night rhythm, a temperature of 21 °C, and a light intensity of 100 µmol m^−2^ s^−1^. After 8 days, the lids of the trays were slightly opened and gradually removed over a two-day-period. Ten-day-old seedlings were transplanted into individual pots filled with autoclaved river sand and potting soil mixture (1:1, *v:v*). Pots were supplied with water from the bottom up three times per week. At an age of 3 weeks, the plants were supplied with half-strength Hoagland solution once a week. Five-week-old plants were used for all experiments. Plants growth was repeated three times independently and each plant set represented one biological replicate.

### 3.2. Metabolite Extraction and LC-MS Analysis

Two hundred milligrams of Col-8 and *glyI4* leaves were finely ground in liquid nitrogen, and powder was used for metabolite extractions. Cells were lysed by thermal shock (freezing/heating). A cold (−20 °C) solution of 60% methanol/40% chloroform was added to each tube. The tubes were mixed for 30 min and subsequently centrifuged at 1000× *g* for 1 min at 4 °C, before being transferred to −20 °C for 2–8 h. After thawing, liquid phases were recovered, and the samples were incubated at 4 °C for 20 min and centrifuged at 13,500× *g* for 10 min at 4 °C; and the collected supernatants were dried to get visible pellets. Lastly, the dried samples were resuspended in water and 5% formic acid, and transferred to glass autosampler vials for LC/MS analysis. Twenty microliters of samples were injected into an Ultra High-Performance Liquid Chromatography (UHPLC) system (Ultimate 3000, Thermo Fisher Scientific, Waltham, MA, USA) and run in positive ion mode. A Reprosil C18 column (2.0 mm × 150 mm, 2.5 μm- Dr Maisch HPLC GmbH, Ammerbuch, Germany) was used for metabolite separation. Chromatographic separations were achieved at a column temperature of 30 °C and flow rate of 0.2 mL/min. A 0–100% linear gradient of solvent A (ddH_2_O, 0.1% formic acid) to B (acetonitrile, 0.1% formic acid) was employed over 20 min, returning to 100% A in 2 min and a 6-min post-time solvent A hold. The UHPLC system was coupled online with a mass spectrometer Q-Exactive (Thermo Fisher Scientific, Waltham, MA, USA) scanning in full MS mode (2 μscans) at 70,000 resolution in the 67 to 1000 m/z range, target of 1 × 10^6^ ions and a maximum ion injection time (IT) of 35 ms. Source ionization parameters were: spray voltage, 3.8 kV; capillary temperature, 300 °C; sheath gas, 40; auxiliary gas, 25; S-Lens level, 45. Calibration was performed before each analysis against positive ion mode calibration mixes (Piercenet, Thermo Fisher Scientific, Waltham, MA, USA) to ensure sub ppm error of the intact mass. Three biological and technical replicates were carried out for both the samples.

### 3.3. Metabolomic Data Processing

#### 3.3.1. Data Elaboration and Statistical Analysis

Raw files of metabolomics data from technical and biological replicates of samples were analyzed using XCMS Online, a freely accessible metabolite database called METLIN (http://metlin.scripps.edu, 10 July 2019) [75]. Raw data sets were uploaded to XCMS Online, and a single group job was created to analyze the difference between profile of *glyI4* mutant versus Col-8. The raw data files were then processed for peak detection, retention-time correction, chromatogram alignment, metabolite feature metadata, and statistical evaluation using the predefined workflow settings for Orbitrap, and metabolite identification was facilitated through METLIN standard database matching and Kyoto Encyclopedia of Genes and Genomes (KEGG) pathway database. XCMS extracted metabolomic features with statistically significant expression changes among the two groups to produce a list of differentially expressed metabolites based on log_2_-fold change > |1| and *p*-value < 0.05.

#### 3.3.2. Pathway Analysis

Metabolite data set were processed using the MetaboAnalyst 5.0 software (http://www.metaboanalyst.ca, 20 October 2021) [76]. The list of metabolites was imported into Metaboanalyst and mapped to the Kyoto Encyclopedia of Genes and Genomes (KEGG) in the “Pathway analysis” tool that supports metabolic pathway analysis, integrating pathway enrichment analysis and pathway topology analysis. Metabolic pathways with *p*-values < 0.05 and pathway impact > 0 were considered significantly enriched. Among these pathways, the top three most significant were widely discussed.

### 3.4. RNA Extraction and RT-qPCR

RNA was isolated from Col-8 and *glyI4* leaves using the “Nucleospin RNA II” kit (Machery-Nagel, Düren, Germany) according to the manufacturer’s instructions. The RNA quality and concentration were estimated by agarose gel electrophoresis and spectrophotometric reading, respectively. DNA contamination was assessed by detecting the absence of amplification bands in no RT samples. cDNA was synthesized using the ImProm-II™ Reverse Transcription System (Promega, Madison, WI, USA) starting from 1 μg of RNA as template and using the oligo-dT primer for first strand synthesis. Diluted cDNA was used as template for Real-Time qPCR with a CFX 96 Touch Real-Time PCR detection system (Bio-Rad, Hercules, CA, USA). Each 10 μL reaction mix contained 5 μL Sso Advanced SYBR Green Supermix (Bio-Rad) and 0.5 μM (final concentration) gene specific primers. Primers were designed with Primer3 software (http://bioinfo.ut.ee/primer3-0.4.0/, 20 May 2019) under default conditions. The amplification program was as follows: 95 °C for 5 s; 40 cycles at 95 °C for 5 s and primers annealing at 54 °C for 45 s. To detect and exclude non-specific amplicons, the melting curves of all PCR products were analyzed (65–95 °C with an increase of 0.5 °C every 5 s). Real-time DNA amplification was processed using CFX Manager™ Software (Bio-Rad). Quantitative analysis was performed according to the 2^−ΔΔCq^ method by applying efficiency correction formula. The normalized amount of target reflects the relative amount of target transcripts with respect to the endogenous reference gene *UBI10*. Gene expression fold change was calculated in *glyI4* mutant relative to Col-8 plants. The Arabidopsis gene identifier (AGI) numbers of the studied genes are AT3g53260 (*PAL2*), AT3g19450 (*CAD4*), AT1g48860 (*EPSPS*), At4g05320 (*UBI10*). Primers were the following: *PAL2* (Fw: AGGCAGCGTTAAGGTTGAGT; Rv: GGTGACTCCGTAACTGTCAGTAC), *CAD4* (Fw: GACACCATGATCGTCAATCAAAAG; Rv: TCAACGGACTATAAACCGTTACTC), *EPSPS* (Fw: TGCTAAATGGTTCTGAGATTCGTC; Rv: AGACCCGAGATTTCTCTAAT GGG), *UBI10* (Fw: AAAGAGATAACAGGAACGGAAACATAGT; Rv: GGCCTTGTAT AATCCCTGATGAATAAG). Results of qPCR analyses were verified by three independent experiments each of them having three technical replicates.

### 3.5. Protein Extraction

One gram of Col-8 or *glyI4* leaves, from plants grown as described in 3.1, were finely ground with mortar and pestle with continuous addition of liquid nitrogen. The powder was resuspended in 5 mL of a cold extraction buffer consisting of 50 mM sodium phosphate buffer (pH 7.5) including 1 mM EDTA, 1% (*w/v*) polyvinylpyrrolidone, 3 mM DTT, and a cocktail of protease inhibitors (Complete ULTRA tablets, Roche, Basel, Switzerland). The homogenate was centrifuged (Universal 32R, Hettich, Tuttlingen, Germany) at 9000 rpm for 15 min at 4 °C and the supernatant was used for enzyme activity assays as described in [77]. The protein content was estimated according to Bradford method using BSA as a standard.

### 3.6. Superoxide Dismutase Activity Assay

SOD activity was assayed with the SOD determination kit (Sigma-Aldrich, Uppsala, Sweden) following the manufacturer’s instructions. The SOD Assay Kit is based on the use of Dojindo’s highly water-soluble tetrazolium salt (2-(4-iodophenyl)-3-(4-nitrophenyl)-5-(2,4-disulfophenyl)-2H tetrazolium, monosodium salt), which produces a water-soluble formazan dye upon reduction with a superoxide anion. The rate of the reduction is linearly related to the xanthine oxidase activity, and it is inhibited by SOD. Since the absorbance at 440 nm is proportional to the amount of formazan dye, the SOD activity can be quantified as an inhibition activity by measuring the decrease in the color development at 440 nm. Thus, inhibition activity corresponds to the amount of total protein extract necessary to reduce the formation of formazan by 50% (IC_50_).

### 3.7. Catalase Activity Assay

CAT activity was measured by monitoring the decrease of absorbance at 240 nm, 25 °C, due to the decomposition of H_2_O_2_ (ε_mM_ = 0.0436 mM^–1^ cm^–1^). The reaction mixture (1 mL final volume) contained 19 mM hydrogen peroxide (H_2_O_2_) in 50 mM potassium phosphate buffer (pH 7.0); the reaction was started by adding 50 μg quantity of protein extract. Catalase specific activity is defined in terms of μmoles of hydrogen peroxide consumed per minute per mg of protein sample.

### 3.8. Thiobarbituric Acid Reactive Substance Measurement

The level of TBARS was used to assess lipid peroxidation following the protocol described in [77]. Briefly, four hundred milligrams of frozen leaves were finely ground using a mortar and pestle under continuous addition of liquid nitrogen. The powder was resuspended in 3 mL of trichloroacetic acid (TCA), 0.1%, and mixed on the vortex until homogenized. Following centrifugation at 13,000 rpm for 10 min, 400 μL of the supernatant (or 0.1% TCA for the blank) was added either to 1 mL of 0.5% TBA in 20% TCA (+TBA solution) or to 1 mL of 20% TCA (–TBA solution) (dilution factor 1:3.5). Samples were incubated at 80 °C for 30 min and then cooled on ice. After centrifugation at 13,500 rpm for 5 min, the absorbance was measured both at 532 nm, which represents the maximum absorbance of the TBA–TBARS complex, and at 600 nm to allow correction of non-specific turbidity. To calculate the TBARS equivalent (nmol mL^–1^), the ε_μM_ (0.155 μM^−1^ cm^−1^) of malondialdehyde (MDA), one of the main products of membrane damage, was used according to the following formula:

[A/ε_μM_ MDA] × dilution factor

where A = [(A_532_nm _(+TBAsol)_ − A_600_nm _(+TBAsol)_) − (A_532_nm _(−TBAsol)_ − A_600_nm _(−TBAsol)_)]

### 3.9. Statistical Analysis

A Mann–Whitney non-parametric test was performed with GraphPad Prism 7.0 (GraphPad Software Inc., San Diego, CA, USA). In detail, the test was applied to analyze differences in PAL2, CAD4, and EPSPS transcript levels in Col-8 and *glyI4* plants (par. 2.2), and to analyze differences in SOD and CAT activity, and T-BARS content in Col-8 and *glyI4* plants (par. 2.3).

## 4. Conclusions

Our current work has shown that metabolic processes linked to redox reactions and the generation of energy sources are upregulated in the *glyI4* mutant compared to Col-8, whereas those involved in defense and growth are downregulated. These findings strongly matched the previously results obtained with the *glyI4* mutant, in which MG rising led to ROS accumulation, reduced growth and fitness, and compromised defense [19]. Lastly, these results have also demonstrated that metabolic profiling gives important information essential to understanding physiological and biochemical rewiring, and ultimately to shedding some light on how these changes affect plant phenotypes. In the future, the awareness of GLYI4 as a novel potential player in the growth–defense trade-off could be exploited to develop sustainable crops.

## Data Availability

The raw metabolomics data are available in the EMBL-EBI MetaboLights database (DOI:10.1093/nar/gkz1019, PMID:31691833) with the identifier MTBLS3724.

## Supplementary Materials

The following are available online at https://www.mdpi.com/article/10.3390/plants10112464/s1, Table S1: All detected and differentially synthesized metabolites in *glyI4* mutant compared to Col-8 (log_2_-fold change > |1|, *p*-value < 0.05).
